# Ultrasound Assessment of Muscle Injury Associated with Closed Limb Fracture

**DOI:** 10.1155/2019/9365291

**Published:** 2019-06-11

**Authors:** Wenfen Liu, Dong Wang, Hui Ouyang, Ningning Chen, Boyang Chang, Qingtang Zhu, Jiachun Li

**Affiliations:** ^1^Department of Ultrasound, The Seventh Affiliated Hospital of Sun Yat-sen University, 628# Zhenyuan Road, Shenzhen, Guangdong 518100, China; ^2^Department of Orthopedic, The Seventh Affiliated Hospital of Sun Yat-sen University, 628# Zhenyuan Road, Shenzhen, Guangdong 518100, China; ^3^Department of Gastroenterology, The Seventh Affiliated Hospital of Sun Yat-sen University, 628# Zhenyuan Road, Shenzhen, Guangdong 518100, China; ^4^Department of Vascular Interventional Radiology, The Third Affiliated Hospital of Sun Yat-sen University, 600# Tianhe Road, Guangzhou, Guangdong 510630, China; ^5^Department of Microsurgery, Orthopedic Trauma and Hand Surgery, The First Affiliated Hospital of Sun Yat-sen University, No. 58 Zhongshan 2nd Road, Guangzhou 510080, China

## Abstract

**Objective:**

The aim of this study was to assess muscle injury associated with upper and lower closed limb fracture using ultrasound, and to develop ultrasound classification criteria for muscle injury.

**Patients and Methods:**

Thirty patients with limb fracture and muscle injury participated in this study. Ultrasonography was used to assess muscle fibre, hematoma, vascular injury, and diameter growth rate. Injury was classified into three grades according to the ultrasound imaging: scores of less than, equal to, or greater than 9.

**Results:**

Of 30 patients, focal fibre rupture was observed in 11 cases; in 9 cases, the injured area exceeded 30% of the muscle area. Six patients had muscle hematoma (the largest reaching 39 mm); in 4 patients, the hematoma showed a honeycombed pattern. Vascular rupture was observed in 6 patients, of which 2 had decreased main arterial diameter and blood flow. The greatest increase in muscle thickness was 17 mm. Of all patients, 11 showed an increase in the diameter growth rate of the muscle exceeding 50%. In addition, among the 30 patients, 11 patients with scores ranging from 4 to 8 received conservative treatment; 9 patients with scores ranging from 10 to 14 received operative treatment; and 10 patients with scores equal to 9 received either conservative or operative treatment.

**Conclusions:**

Ultrasonography is useful for diagnosing muscle injury associated with closed limb fracture. The ultrasound classification criteria for muscle injury can be used to assess the severity of injury and guide the decision of treatment.

## 1. Introduction

Muscle injury associated with closed limb fracture is commonly observed in daily medical practice. Although clinical evaluation remains the mainstay of the early and accurate diagnosis of muscle injury, posttraumatic local oedema and severe pain can limit physical examination, and complete muscle tears can even be missed. However, an early image diagnosis can reduce patient discomfort and guide the decision of whether to implement surgical or conservative treatment [1].

The muscle is a soft tissue that is most easily studied by ultrasonography. Moreover, ultrasonography has been one of the first imaging techniques available for the evaluation of muscle disease [2–4]. Due to advances in ultrasound technology, it offers significant advantages over other imaging techniques in assessing muscle injury [5]. Owing to its multiplanar approach, both transversal and longitudinal, dynamic examination of muscle, excellent spatial resolution, and definition of muscle structure, ultrasonography is on the leading edge in the assessment of muscle pathology [1, 2, 5, 6]. Furthermore, it is faster, more convenient, and cheaper than magnetic resonance imaging (MRI). However, at present, muscle ultrasonography is mainly used in sports traumatology to help a physician decide whether a patient should or should not resume professional training and competition [2, 7]. The use of ultrasonography to examine limb fracture associated with muscle injury is uncommon.

When closed limb fracture is associated with muscle injury, it is important to assess the viability of the muscle to decide whether a surgery is required, and which area should be operated on. In our study, we used ultrasonography to examine muscle pathology and, at the same time, to assess the viability of the muscle in limb fracture associated with muscle injury. We focused our ultrasound assessment on 4 characteristics of muscle injury—muscle fibre [2, 7–9] and complications of muscle rupture, namely, muscle hematoma, vascular injury [10], and diameter growth rate of the muscle [5, 11–15]—to develop the ultrasound classification criteria for muscle injury.

## 2. Materials and Methods

The study was approved by the Ethics Committee at the Seventh Affiliated Hospital of Sun Yat-sen University. All procedures were carried out in accordance with ethical standards, and informed consent was obtained from all patients.

This study was performed using color ultrasound on patients with acute muscle injury associated with closed limb fracture. A total of 30 patients with limb fracture and muscle injury were examined with ultrasound within 24 hours after fracture and before surgery.

In our study, we only examined muscle pathology related to limb fractures and excluded other conditions such as chronic lesions, sports injuries, muscle inflammation or infection, muscle hernia, muscle tumour, or intrinsic injury.

Muscles are usually examined using a linear array high-frequency transducer [16]. In our study, we used a 7-MHz to 15-MHz compact linear array ultrasound transducer (Venue 40, General Electric Health, Schenectady, NY, USA) to examine muscle injury associated with closed limb fracture. With this machine, we used color-flow ultrasound imaging, which is widely used to show the blood flow in main arterial or venous injury [17] and to help delineate the areas of acute muscle injury by showing increased blood flow to the affected areas [16].

First, we used color ultrasound to assess the 4 major characteristics of muscle injury: muscle fibre and complications of muscle rupture, namely, muscle hematoma, main vascular injury, and diameter growth rate of the muscle. To measure the diameter growth rate of the muscle (*R*_*change*_), we first measured the maximum thickness of the muscle from the superficial to deep layer of the muscle in both the injured (*F*_*injury*_) and uninjured limbs (F_*control*_). Then, we calculated the diameter using the following equation: *R*_*change*_ = (*F*_*injury*_ − F_control_)/F_control_. Next, we compared the results with operative or pathological findings. Last, each of the four characteristics was given a score to represent the degree of severity. In addition, injury was classified into 3 grades according to the ultrasound imaging: score less than 9, equal to 9, and more than 9.

## 3. Results

The ages of the patients ranged from 15 to 75 years (mean age was 32.8 years). There were 18 men and 12 women. Seventeen patients had upper limb injury and the other 13 had lower limb injury. Focal fibre rupture was observed in 11 cases; in 9 cases, the injured area exceeded 30%. Sixteen patients had muscle hematoma (the largest reaching 39 mm); in 4 patients, the hematoma showed a honeycombed pattern. Vascular rupture was observed in 6 patients, of which 2 had decreased arterial diameter and blood flow. The greatest increase in muscle thickness was 17 mm. Of all patients, 11 showed an increase in the diameter growth rate of the muscle exceeding 50%, and 11 showed an increase in the area growth rate of the muscle exceeding 50%.

### 3.1. Ultrasonography of Muscle Fibre

The main goal of ultrasonography in muscle injury associated with limb fracture is to assess the viability of the injured muscle. In muscle injury, the lesions can be divided using the four-grade classification system as recommended previously by numerous authors, including us [2, 7, 8].

**Figure 1:**
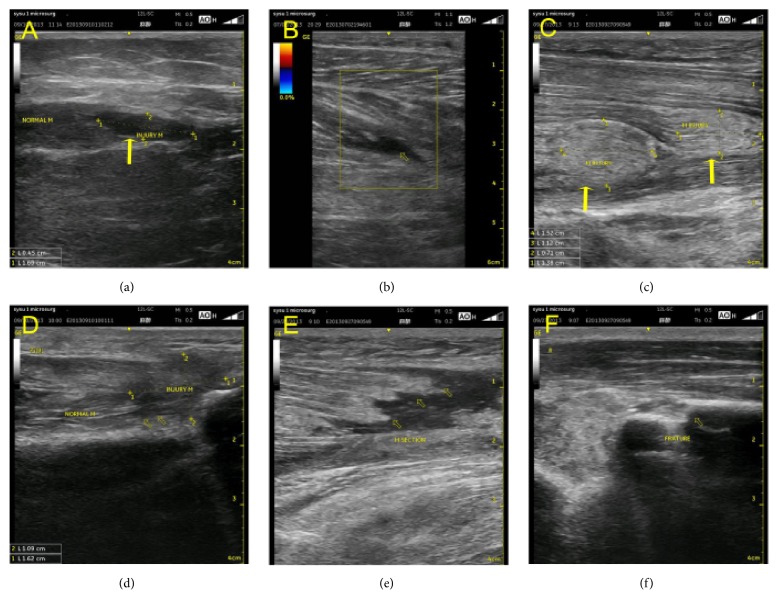
Grade I muscle rupture, but focal fibre rupture with less than 5% of the muscle involved; ultrasonography shows a hypoechoic mass within the muscle fibres (yellow arrow) (a, b). Grade II partial muscle rupture; the muscle belly forms a real mass, with focal fibre rupture with more than 5% but less than 30% of the muscles involved. Ultrasonography shows a hypoechoic mass within the muscle fibres (yellow arrow) (c, d). Grade III lesion represents focal fibre rupture with more than 30% of muscle rupture with retraction, and it also shows the bell-clapper sign, with torn muscle fragments floating in the serohematic fluid (yellow arrow) (e). Ultrasonography shows a limb fracture, fracture of the radius (yellow arrow point) (f).

Grade 0 corresponds to no lesions or, in other words, no abnormal areas, on ultrasound examination. Grade I corresponds to focal fibre rupture with less than 5% of the muscle involved [2]. However, in many cases, Grade 0 cannot be clearly distinguished from Grade I. Therefore, Grades 0 and I can be merged into a single grade—Grade I—characterized by no abnormalities or focal fibre rupture with less than 5% of the muscle involved [7, 18] (Figures 1(a) and 1(b)).

Grade II corresponds to partial muscle rupture, which is focal fibre rupture with more than 5% but less than 30% of the muscle involved with or without fascial damage [7, 8, 18, 19]. Ultrasonography can often clearly demonstrate a hypoechoic or even anechoic gap within muscle fibres (Figures 1(c) and 1(d)). When using the ultrasound transducer with gentle pressure, we can see torn muscle fragments floating in a serohematic fluid. This causes a peculiar sign, known as the bell-clapper sign, as shown in Figure 1(e). The bell-clapper sign is used to describe the retracted echogenic muscle fragments surrounded by hypoechoic hematoma [2, 5].

Grade III lesions are characterized by focal fibre rupture of more than 30% to complete muscle rupture with retraction and can be associated with fascial damage. Grade III lesions may be observed in patients with severe muscle rupture associated with limb fracture, although they are uncommon. Sometimes a few lesions are clinically evident as grade III lesions because the muscle belly forms a real mass or because a gap can be palpated between the retracted ends of the muscle (Figures 1(c), 1(d), and 1(e)) [2].

The three grades correspond to progressively increasing severity of muscle rupture or injury in patients with limb fracture. When muscle injury is associated with limb fracture, a tendency may be observed, in which the higher the grade of the muscle rupture, the lower the degree of the muscle injury. In some cases of superficial limb fracture, we can also observe the fracture on ultrasonography (Figure 1(f)).

### 3.2. Major Early Complication of Muscle Rupture: Muscle Hematoma

**Figure 2:**
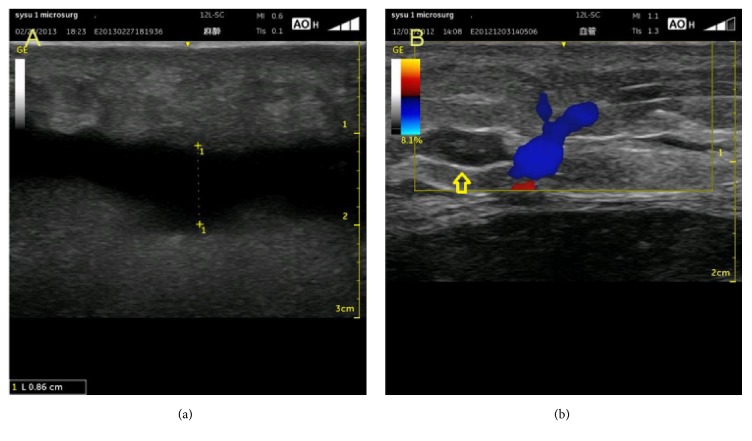
Hematoma presenting as a hypoechoic (b) or anechoic (a) circumscribed lesion.

Muscle hematoma, both intramuscular and intermuscular, is the major early complication of muscle ruptures [20] and is another key ultrasound feature of muscle injury. In most of the cases, the hematoma presents as a hypo- or anechoic circumscribed lesion (Figures 1(e) and 2(b)). However, in severe cases, some lesions may have a honeycombed patternon ultrasonography (Figure 5(a)) probably because the injured muscle becomes swollen and numerous small hematomas form in the muscle [5].

Based on the above, we can distinguish 3 grades depending on the severity of the rupture. The first grade is no hematoma or one small hematoma (diameter <10 mm, including either intramuscular or intermuscular hematoma); the second grade is from 1 to 2 hematomas (total diameter: 10–30 mm); and the third grade is 3 or more hematomas (total diameter: >30 mm) or a honeycombed pattern shown on ultrasound.

In some cases of severe muscle ruptures, the hematoma can be quite large (Figures 2(a) and 5(b)), resulting in compartment syndrome that will further compromise the condition of the surrounding muscles, vessels, and nerves. In these cases, a needle evacuation of the hematoma may be recommended [2, 19].

### 3.3. Vascular Injury on Ultrasonography

**Figure 3:**
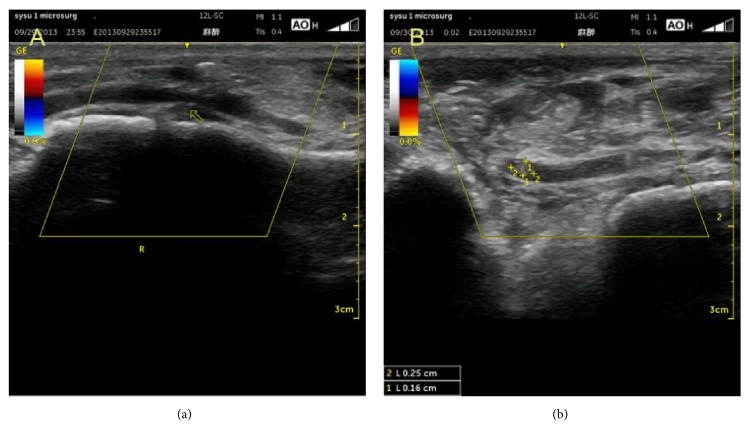
Vascular abnormalities on ultrasonography: decreased arterial diameter and blood flow (a); partial arterial or venous disruption, partial arterial thrombosis (b).

Color Doppler ultrasound imaging is the most useful method for evaluating blood flow in the skeletal muscle. A positive Doppler signal and color ultrasound imaging allow detecting even minor strain injuries (Figures 3(a) and 3(b)). In ultrasound imaging of the muscle injury, there are 4 features of the main vascular injury that need to be considered, namely, partial or complete arterial or venous disruption, main artery thrombosis, decreased arterial diameter, and decreased arterial blood flow.

To identify the severity of vascular damage in muscle injury, we distinguished 3 grades. Grade 1 corresponds to no abnormalities on vascular ultrasonography. Grade 2 corresponds to a few abnormalities on vascular ultrasonography, including a decreased arterial diameter and blood flow, partial arterial or venous disruption, partial arterial thrombosis, and main artery thrombosis. Grade 3 corresponds to severe abnormalities on vascular ultrasonography, such as complete arterial rupture, complete artery thrombosis, and absence of blood flow in the distal muscle.

The different grades of vascular damage correspond to various degrees of muscle injury, and patients may generally have a poor long-term outcome if the artery shows irreversible severe arterial damage [10].

### 3.4. Diameter Growth Rate of Muscle

When assessing muscle injury, another important aspect is an increase in the muscle diameter due to contusion and swelling of the injured muscle [5, 11–14]. Intramuscular contusion occurs after blunt trauma to the muscle and presents with immediate and prolonged pain at the injury site. On ultrasonography, a contusion is seen as an ill-defined, hyperechogenic area in the muscle that may cross the fascial boundaries. In acute cases, the muscle becomes swollen and may be isoechoic with an uninjured muscle [5, 15].

**Figure 4:**
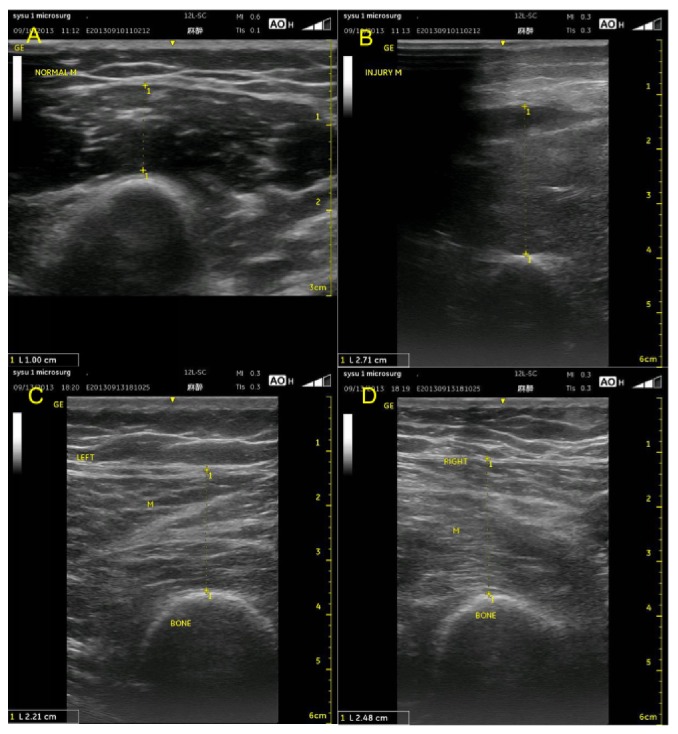
The diameter growth rate of the muscle is 170%, and this group can be classified as Grade 3; the growth rate exceeds 50% ((a) and (b)). The diameter growth rate of the muscle is 12%, and this group can be classified as Grade 2; the growth rate is between 10% and 50% ((c) and (d)).

**Figure 5:**
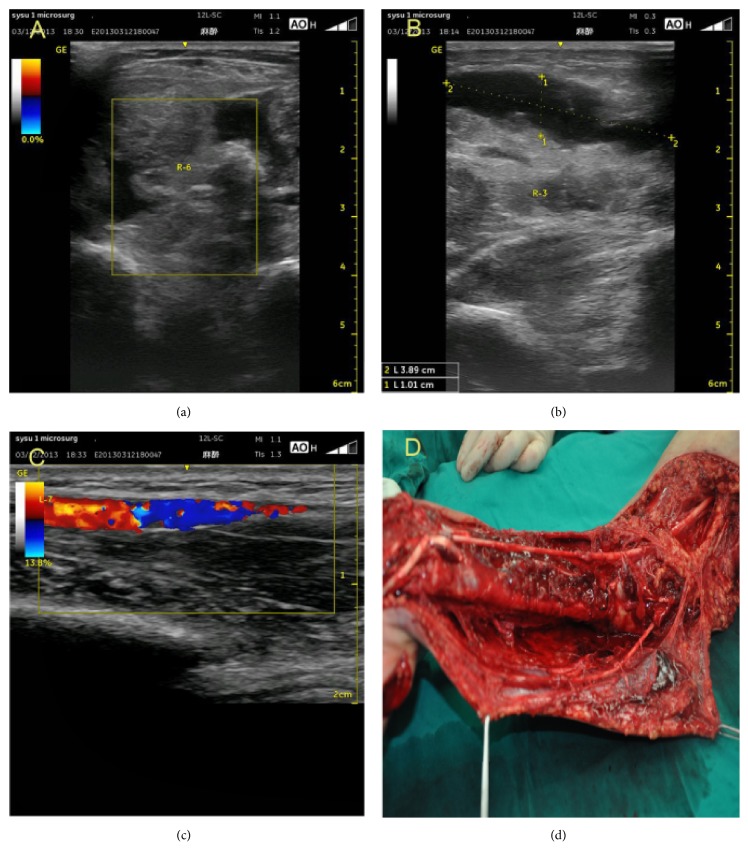
Ultrasound classification criteria for muscle injury—a case with a total score of 13 points. (a) Grade III lesions, complete muscle fibre rupture, ruptured muscle with retraction, fascial injury, the bell-clapper sign, torn muscle fragments floating in the serohematic fluid. (b) Hematoma presenting as an anechoic circumscribed lesion; the total muscle diameter is 39 mm. (c) Decreased arterial diameter and blood flow, partial arterial disruption. (d) The result of this patient after surgery: complete muscle fibre rupture, fascial injury, several hematomas present, partial but continuous arterial disruption.

In our patients, the diameter growth rate of the muscle was from 0% to 170%, and we used 3 grades to distinguish between those rates. Grade 1 corresponds to a growth rate of less than 10%; Grade 2, 10% to 50% (Figures 4(c) and 4(d)); and Grade 3, more than 50% (Figures 4(a) and 4(b)). These 3 grades correspond to different degrees of muscle injury.

## 4. Discussion

Muscle injuries are the most common injuries in patients with closed limb fracture. Recently, an increasing number of orthopedic surgeons have started to consider muscle injury as more important than limb fracture itself, because it is closely related to the functional recovery of the upper limb and lower limb. Although clinical evaluation remains the mainstay of the early and accurate diagnosis of a muscle injury, posttraumatic local oedema and severe pain can limit physical examination, and even complete muscle tears can be missed. Therefore, an early diagnosis can reduce patient discomfort and guide the decision of whether to implement surgical or conservative treatment [1].

There are two diagnostic imaging methods, ultrasonography and MRI, that can be used for an early diagnosis of muscle injury. Compared with ultrasonography, MRI seems to be superior for diagnosing and predicting the outcome of patients with muscle injuries [20]. The MRI measurements of the lesion size, including height on longitudinal sections above the cross-sectional surface, may be useful [21]. However, the available data are limited and contradictory, particularly regarding the influence of the initial lesion size on recovery time and recurrence risk after acute hamstring strains [22–24]. Recent advances in ultrasound technology have allowed clinicians to obtain detailed images, which are as accurate as those obtained by MRI, for the evaluation of muscle injuries [20, 25]. Muscles are among the soft tissues that are most easily studied by ultrasound examination. Moreover, it was the first imaging method available for the evaluation of muscle disease [2]. With the advances in ultrasound technology, it offers significant advantages over other imaging techniques in assessing muscle trauma [5]. Owing to its multiplanar approach, both transversal and longitudinal, dynamic examination of the muscle, excellent spatial resolution, and definition of muscle structure, ultrasonography is on the leading edge when it comes to assessing muscle pathology [1, 2, 5, 6]. Furthermore, ultrasonography is faster, more convenient, and cheaper than MRI. A prospective study [26] showed that ultrasonography was as useful as MRI for assessing acute hamstring injury.

In this study, we used ultrasonography to assess muscle fibre rupture [2, 7, 8], muscle hematoma [2, 7], main vascular injury [10], and diameter growth rate of the muscle [5, 11–15, 27]. The ultrasound assessment of muscle fibre rupture directly reflects the muscle injury; muscle hematoma is one of the most important complications of muscle rupture; ultrasound assessment of main vascular injury shows blood flow in the injured muscle; and the diameter growth rate of the muscle reflects the extent of contusion and swelling. These four aspects are the most important when assessing muscle injury associated with upper and lower limb fracture.

Based on the above four characteristics of muscle injury, we used the existing clinical assessment standards, such as the mangled extremity syndrome index [28] and the mangled extremity security score [29], as well as the available data on ultrasound assessment of muscle injury [1, 2, 5, 7, 8, 10, 19, 24, 25, 30–32] to establish the ultrasound classification criteria for muscle injury (Table 1). Muscle fibre, which is the first ultrasound classification criterion, is considered to be the most important one because it directly reflects the changes of the injured muscle. The other criteria (B: complication of muscle rupture (muscle hematoma); C: main vascular injury; D: diameter growth rate of the muscle) score the same amount of points because, in our opinion, they equally contribute to early muscle injury associated with limb fracture. The total score in the classification criteria is 14 points, and the patients may gain a score from 4 to 14 points. Depending on the score achieved by a patient, a different treatment is administered. Eleven patients with scores ranging from 4 to 8 received conservative treatment; 9 patients with scores ranging from 10 to 14 received operative treatment; and 10 patients with scores equal to 9 received either conservative or operative. Thus, patients with a score of less than 9 points may have muscle injury that is not severe, and surgical treatment should be applied in patients scoring greater than 9 points (fasciotomy, blood vessel or nerve exploration, removal of the necrotic tissue and hematoma, incision decompression, osseous fascia compartment) (Table 2). Similarly, the classification criteria may be used to predict prognosis. If the score is less than 9, the prognosis is likely to be good, and when the score is greater than 9, the prognosis is likely to be poor. However, in order to improve further studies, we should have a greater sample size of patients.

In conclusion, ultrasonography is useful for diagnosing acute muscle injury associated with limb fracture. The ultrasound classification criteria for muscle injury can be used to predict the severity of injury and guide decision on the type of treatment. However, the criteria needs to be verified on a larger sample of patients, and some sections of the proposed criteria may require a more detailed analysis.

## Figures and Tables

**Table 1:** Ultrasound classification criteria for muscle injury.

Type	score
*(A) Muscle fibre*	
*Grade I:*	1
No abnormalities, with/without focal fibre rupture with less than 5% of the muscle involved	
*Grade II:*	3
Partial rupture: focal fibre rupture with more than 5% but less than 30% of the muscle involved with/without fascial damage	
*Grade III:*	
Focal fibre rupture with more than 30% to complete muscle rupture with retraction, fascial damage	5
*(B) Complication of muscle rupture: muscle haematoma*	
*Grade I:*	
No haematoma or small haematoma (total diameter <10 mm)	1
*Grade II:*	
1–2 haematomas (total diameter: 10–30 mm)	2
*Grade III:*	
3 or more haematomas (total diameter: >30mm), or a honeycombed pattern	3
*(C) Main vascular injury *	
*Grade I:*	
No abnormalities on vascular ultrasonography	1
*Grade II:*	
Decreased arterial diameter and blood flow, partial arterial or venous rupture, partial arterial thrombosis	2
*Grade III:*	
Complete arterial rupture, complete arterial thrombosis and no blood flow in the distal muscle	3
*(D) Diameter growth rate of the muscle (scored in the maximum number) *	
*Grade I: *Growth rate <10%	1
*Grade II: *Growth rate 10%–50%	2
*Grade III: *Growth rate >50%	3
*Total score*	14

**Table 2:** Patients distribution in four types and score.

Type	A		B		C		D	
	Grade I	19	Grade I	24	Grade I	24	Grade I	9
	Grade II	2	Grade II	2	Grade II	4	Grade II	10
	Grade III	9	Grade III	4	Grade III	2	Grade III	11

Score<9	11		Score=9	9		Score>9	10	

## Data Availability

All data needed to evaluate the conclusions in the paper are present in the paper and in the references cited. Additional data related to this paper may be requested from the authors.
